# The Combination of CD147 and MMP-9 Serum Levels Is Identified as Novel Chemotherapy Response Markers of Advanced Non-Small-Cell Lung Cancer

**DOI:** 10.1155/2020/8085053

**Published:** 2020-04-24

**Authors:** Xiaojuan Qiao, Yan Gu, Jingfeng Yu, Jinghui Wang, Xuan Liu, Meng Gu, Li Ma, Yongfeng Jia, Shucai Zhang

**Affiliations:** ^1^Department of Medical Oncology, The Affiliated Hospital of Inner Mongolia Medical University, Hohhot, Inner Mongolia, China; ^2^Department of Respiratory Medicine, The Affiliated Hospital of Inner Mongolia Medical University, Hohhot, Inner Mongolia, China; ^3^College of Basic Medicine, Inner Mongolia Medical University, Hohhot, Inner Mongolia, China; ^4^Department of Medical Oncology, Beijing Chest Hospital, Capital Medical University, Beijing, China; ^5^Department of Surgery, The Affiliated Hospital of Inner Mongolia Medical University, Hohhot, Inner Mongolia, China; ^6^Department of Cellular and Molecular Biology, Beijing Chest Hospital, Capital Medical University, Beijing, China; ^7^Department of Cellular and Molecular Biology, Beijing Tuberculosis and Thoracic Tumor Research Institute, Beijing, China; ^8^Department of Pathology, The Affiliated Hospital of Inner Mongolia Medical University, Hohhot, Inner Mongolia, China

## Abstract

To evaluate the correlation between the changes in serum concentrations of cluster of differentiation-147 (scCD147) and chemotherapy outcome in patients with NSCLC and evaluate the combination of scCD147 with serum matrix metalloproteinase-9 (scMMP-9) levels in the prediction of chemotherapy response, eighty-two patients with advanced LC were enrolled. Newly diagnosed cases were treated with platinum-based chemotherapy. We measured scCD147 protein levels in LC cases by ELISA and used receiver operating characteristic (ROC) curves to analyze the results. Four time points were chosen to examine the association between the changes in scCD147 and chemotherapy outcome: before chemotherapy and 21 days after the start of the first, second, and fourth chemotherapy cycles. We assessed the combination of scCD147 and scMMP-9 serum levels in predicting the chemotherapy response. scCD147 was higher in LC cases than that in healthy volunteers (HVs). scCD147 was associated with distant metastases and TNM stage. scCD147 and scMMP-9 appeared to be independent predictive factors for chemotherapy outcomes after the first and second chemotherapy cycles for patients with NSCLC. Multivariable analysis also demonstrated that variations in scCD147 and scMMP-9 could be independent factors for monitoring chemotherapy outcome for patients with NSCLC. Furthermore, when scCD147 and scMMP-9 are combined into a new risk model, it has a markedly better prediction of chemotherapy outcomes than each protein alone. scCD147 and MMP-9 are potential predictive biomarkers for efficacy, and their combination significantly improves the predictive power for chemotherapy response in patients with NSCLC.

## 1. Introduction

Lung cancer (LC) is the fifth leading cause of death in China [1]. LC can be of two main types: non-small-cell lung cancer (NSCLC) or small-cell lung cancer (SCLC) [2]. Overall survival at 5 years for LC has improved only slightly over the past 40 years [3]. The treatment is still challenging because of the invasion/metastases of tumor cells [4]. Although surgical resection sometimes cures early stage NSCLC, few therapeutic options are available for advanced-stage NSCLC, highlighting the importance of a better understanding of the disease to find novel therapeutic targets. Therefore, the identification of novel, predictive, and efficacious predictive biomarkers can help to deliver “individualized and efficient” therapy.

Several biomarkers have been found to be involved in the invasion/metastases of tumor cells [5]. Cluster of differentiation (CD) 147 (also known as “extracellular matrix metalloproteinase inducer” or “basigin”) is a member of the immunoglobulin superfamily and was first observed on the surfaces of tumor cells [6, 7]. CD147 is closely associated with the invasion/metastases of tumor cells [8]. With regard to the heterogeneity of tumor microenvironments, CD147 can activate matrix metalloproteinases (MMPs), degrade the extracellular matrix (ECM) of tumor cells, and induce the invasion/metastases of tumor cells [9, 10]. CD147 also plays a pivotal role in inhibiting the apoptosis of tumor cells [11].

MMP-9 has been reported to play a crucial part in the invasion/metastases of tumor cells. MMP-9 can degrade type-IV collagen (which constitutes the ECM and basal membrane). Scholars have postulated that CD147 and MMP-9 could be unique biomarkers for type-II/III astrocyte-elevated genes and predict tumor progression and prognosis. However, the possibility of utilizing serum CD147 and MMP-9 levels as biomarkers has not been validated.

Previously, we showed that serum concentrations of MMP-9 in NSCLC cases during chemotherapy were intimately associated with chemotherapy outcome [12]. Analysis was performed based on the same dataset with the same methods as the previous publication. We evaluated the correlation between the changes in serum concentrations of CD147 and chemotherapy outcome in NSCLC cases and analyzed the relationship with the changes in serum concentrations of MMP-9. In this way, we assessed the possible role of the combination of serum CD147 and MMP-9 levels as biomarkers for evaluating chemotherapy outcomes.

## 2. Materials and Methods

### 2.1. Patients

Eighty-two cases (59 (72%) males and 23 (28%) females; median age, 60 (range, 20–79) years) with advanced LC admitted to Beijing Chest Hospital (Capital Medical University, Beijing, China) from August 2013 to October 2014 were enrolled. The diagnosis was confirmed by histology or cytology. The patients did not receive treatment before study commencement.

Cases were categorized as “smokers” (65 (79.3%)) or “nonsmokers” (17 (20.7%)). Additionally, 72 (87.8%) cases had an Eastern Cooperative Oncology Group Performance Status (ECOG PS) score of 0–1, whereas 10 (12.2%) cases had an ECOG PS score of ≥2. The number of patients with adenocarcinoma, squamous cell carcinoma (SCC), or SCLC was 35 (42.7%), 18 (21.9%), and 29 (35.4%), and those with type-IIIa, type-IIIb, and type-IV disease totaled 8 (9.8%), 17 (20.7%), and 57 (69.5%), respectively.

In 25 (30.5%) cases, metastases had not occurred, whereas in 57 (69.5%) individuals, distant metastasis had occurred. The number of patients with N0, N1, N2, and N3 stages was 8 (9.8%), 3 (3.7%), 23 (28%), and 48 (58.5%), whereas the number with T1, T2, T3, and T4 stages was 3 (3.7%), 28 (34.1%), 13 (15.9%), and 38 (46.3%), respectively. Analysis was performed based on the same dataset with the same methods as our previous publication [12]. This study was approved by the Ethics Committee, and all of the patients provided written informed consent.

Among 30 healthy volunteers (HVs), 18 (60.0%) were male and 12 (40.0%) were female. The median age was 57 (range, 26–68) years. Compared with 82 cases of LC, sex (*P* = 0.227) and age (*P* = 0.951) were not significantly different.

### 2.2. Chemotherapy and Assessment of Response

All cases received platinum-based treatment: cisplatin (75 mg/m^2^) on day 1 and day 2, carboplatin area under curve 5 (300-350 mg/m^2^) on day 2, and nedaplatin (75 mg/m^2^) on day 1. Different histologic types were treated with platinum and other drugs: paclitaxel liposomes (150–175 mg/m^2^ on day 1) or pemetrexed (500 mg/m^2^ on day 1) for adenocarcinoma and paclitaxel liposomes for SCC. The cycle was repeated every 21 days. We excluded patients if they had progressive disease (PD), if unbearable toxicity during chemotherapy was recorded, or if patients refused treatment.

Tumor diameter was measured using computed tomography before chemotherapy as well as 21 days after the start of the first, second, and fourth cycles of chemotherapy. The responses of patients were analyzed by the Response Evaluation Criteria in Solid Tumors based on a complete response (CR), partial response (PR), stable disease (SD), and PD.

The efficacy evaluation was divided into disease control (CR/PR/SD) and progressive disease (PD). In NSCLC patients, there were 47 cases with CR/PR/SD and 6 cases with PD after the first cycle of chemotherapy. After the second cycle of chemotherapy, there were 37 cases with CR/PR/SD, 13 cases with PD (7 cases of PD were newly added in the second cycle), and 3 cases of shedding patients. After the fourth cycle of chemotherapy, there were 14 cases with CR/PR/SD, 25 cases with PD (12 cases of PD were newly added in the cycle), and 11 cases of shedding patients.

### 2.3. Measurement of Serum Concentrations of CD147

Blood samples were obtained before treatment for all patients. For patients with NSCLC, samples were collected at the four time points mentioned above. Serial samples were collected from 53 cases at the first and second cycles, from 44 at the third cycle, and from 26 at the fourth cycle. Each blood sample was centrifuged at 1509 g/min for 5 min at room temperature, separated into plasma and supernatant, and stored at −80°C. Serum concentrations of CD147 were detected using an enzyme-linked immunosorbent assay (ELISA) kit based on the manufacturer's (USCN Life Science, Houston, TX, USA) protocols.

### 2.4. Statistical Analyses

Statistical analyses were performed using SPSS v19.0 (IBM, Armonk, NY, USA). The chi-squared (*χ*^2^) test was carried out to ascertain the relationship between classification variables. Nonparametric statistical analyses were employed due to the broad data range. Serum levels of CD147 are given as median values and ranges. The Mann–Whitney *U*-test was employed to analyze the difference between measurement parameters. A receiver operating characteristic (ROC) curve was created by SPSS v19.0. All tests were two-sided, and statistical significance was set at *P* < 0.05.

For sensitivity and specificity analysis, the following score model was constructed: sensitivity% = (true‐positive rate)/(true‐positive rate + false‐negative rate)∗100%; specificity% = (true‐negative rate)/(true‐negative rate + false‐positive rate)∗100%.

The multivariate Cox proportional hazards regression model was used to evaluate independent factors associated with tumor response. As a multivariate analysis, all clinical factors were included in the model.

## 3. Results

### 3.1. Serum Concentrations of CD147 in LC Patients and HVs

**Figure 1:**
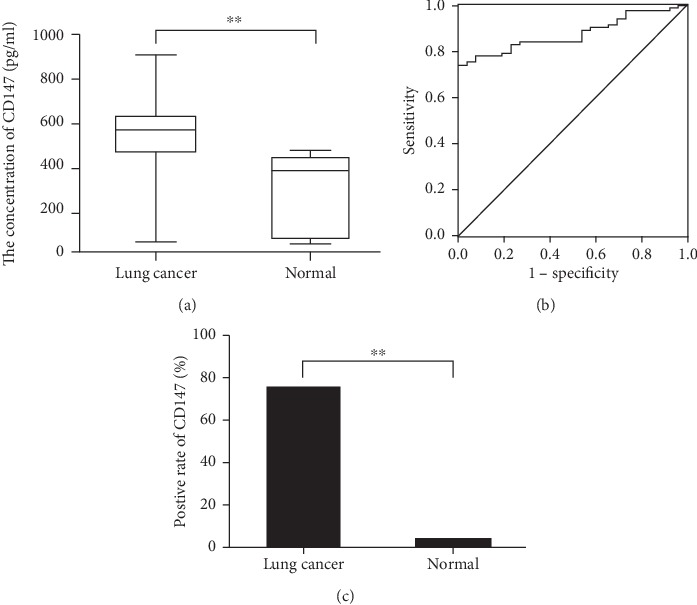
(a) Differences in the serum concentrations of CD147 between patients with lung cancer (LC) and healthy volunteers (HVs). (b) The diagnostic value of serum concentrations of CD147 for identifying LC in a receiver operating characteristic curve. (c) Differences in the positive rate of serum concentrations of CD147 between patients with LC and HVs. ^∗∗^*P* < 0.01.

Serum concentrations of CD147 in 82 LC cases and 30 HVs were measured by ELISA. The mean serum level of CD147 in LC cases was 563.77 (range, 50.32–910.80) pg/mL, which was much higher than that in HVs (381.05 (range: 43.38–474.52) pg/mL) (*P* < 0.001) (Figure 1(a)). ROC curves were employed to ascertain the serum concentrations of CD147. The area under the ROC curve (AUC) was 0.876 (95% confidence interval (CI): 0.812–0.939). The difference in the AUC was significant compared with 0.5 (*P* < 0.001). When the cut-off value for the serum concentration of CD147 was 466.14 pg/mL, the sensitivity was 75.6%, the specificity was 96.2%, the positive predictive value was 98.4%, and the negative predictive value was 59.1% for the diagnosis of lung cancer (Figure 1(b)). Using a serum concentration of 466.14 pg/mL as the cut-off point, 62 (75.6%) LC cases had a higher serum level of CD147, whereas only 1 (3.3%) HV did, and this difference was significant (*P* < 0.001) (Figure 1(c)).

### 3.2. Correlation between the Serum Concentrations of CD147 and MMP-9 before Chemotherapy and Clinical Characteristics in NSCLC

The serum concentrations of CD147 in patients with LC were related to distant metastases and tumor-node-metastasis (TNM) stage. We did not find a significant association between the serum concentrations of CD147 and sex, age, smoking status, ECOG PS score, histology, diameter of primary tumor, or metastases to regional lymph nodes (*P* > 0.05). Serum concentrations of CD147 were significantly higher in cases with the M1 stage than concentrations in cases with the M0 stage (*P* = 0.037). Serum concentrations of CD147 in cases with stage-IV disease were significantly higher than those in cases with stage-III disease (*P* = 0.037).

There was no significant association between the serum concentrations of CD147 in patients with NSCLC and the clinical characteristics (*P* > 0.05) (Table 1). The serum MMP-9 levels before chemotherapy were not significantly associated with the clinical characteristics of the patients with NSCLC [12].

### 3.3. Association between the Changes in Serum Concentrations of CD147, MMP-9, and Tumor Responses in NSCLC

**Figure 2:**
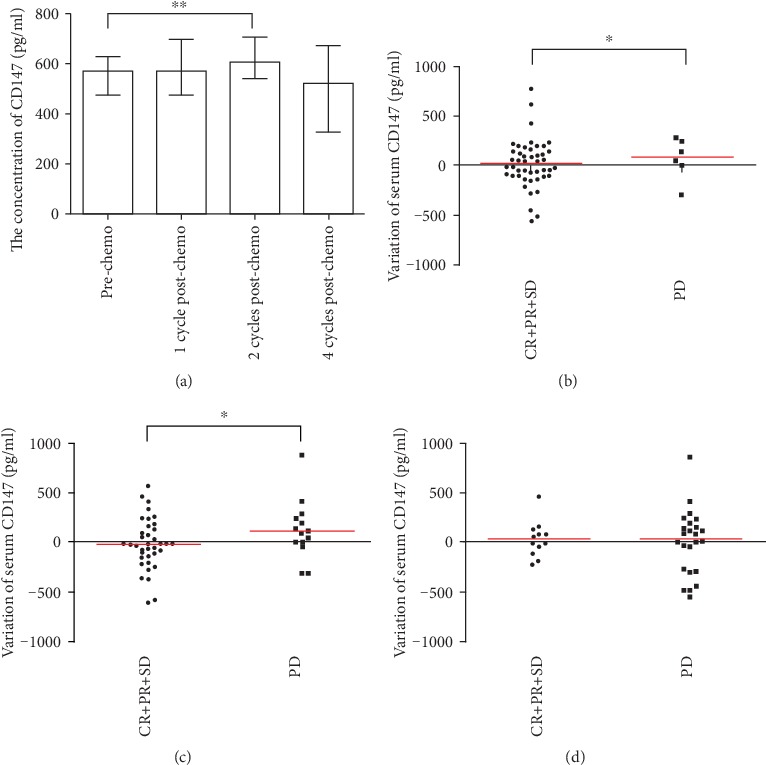
(a) Compared with baseline, serum concentrations of CD147 increased gradually after the first and second chemotherapy cycles and then decreased after the fourth cycle in patients with NSCLC. (b) Association between the changes in serum concentrations of CD147 from baseline to the first chemotherapy cycle and tumor response after the first chemotherapy cycle. (c) Association between the variation in serum concentrations of CD147 from the first to second chemotherapy cycle and tumor response after the second chemotherapy cycle. (d) Association between the alterations in serum concentrations of CD147 from the second to fourth chemotherapy cycle and tumor response evaluated after the fourth chemotherapy cycle. Each dot represents a patient, and red lines represent median values. CR: complete response; PR: partial response; SD: stable disease; PD: progressive disease. ^∗∗^*P* < 0.01 and ^∗^*P* < 0.05.

Serum concentrations of CD147 increased gradually after the first and second chemotherapy cycles and decreased after the fourth cycle (before chemotherapy *vs.* after the first chemotherapy cycle (*P* = 0.389), after the second chemotherapy cycle (*P* = 0.017), and after the fourth chemotherapy cycle (*P* = 0.444)) (Figure 2(a)).

According to the chemotherapy response, we evaluated the difference in alterations in serum concentrations of CD147 in patients with different responses after the first, second, and fourth chemotherapy cycles. We assessed the changes in serum concentrations of CD147 in cases with PD and those with CR/PR/SD.

Compared with before PD, serum concentrations of CD147 increased markedly in patients with PD. Compared with the previous time point of blood collection of cases with CR/PR/SD, serum concentrations of CD147 increased slightly or declined in CR/PR/SD patients. The disparity of alterations in serum concentrations of CD147 in patients with different chemotherapy outcomes was significant after the first (*P* = 0.047) (Figure 2(b)) and second chemotherapy cycles (*P* = 0.036) (Figure 2(c)). However, there were no significant alterations in the serum concentrations of CD147 between cases who achieved PD and those with CR/PR/SD after the fourth chemotherapy cycle (*P* = 0.815) (Figure 2(d)).

The changes in serum concentrations of MMP-9 and tumor responses in NSCLC cases were performed with the same methods as our previous publication [12]. In PD patients, the serum level of MMP-9 increased before PD, and in CR/PR/SD patients, the serum level of MMP-9 was decreased or stable. The difference in variation in serum MMP-9 levels in patients with different chemotherapy curative effects was statistically significant after one cycle, two cycles, and four cycles (after one cycle: *P* < 0.001, after two cycles: *P* < 0.001, and after four cycles: *P* = 0.01) [12].

### 3.4. The Combination of scCD147 and scMMP-9 as a Predictive Model for Chemotherapy Response

**Figure 3:**
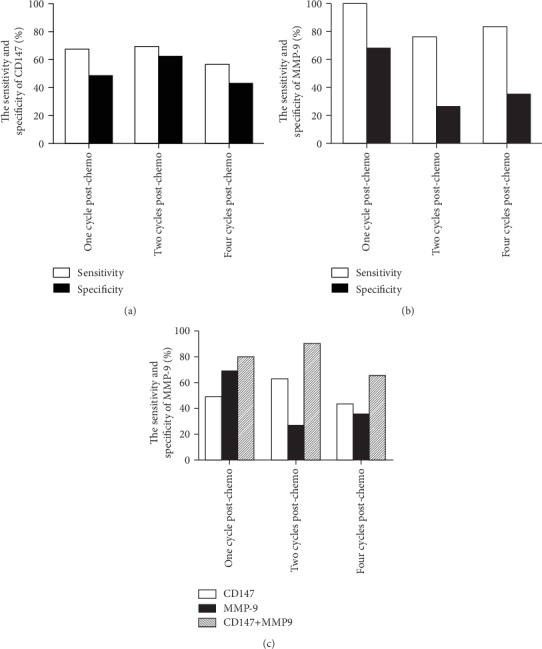
The combination of scCD147 and scMMP-9 as a predictive model for chemotherapy response. (a) The sensitivity and specificity of scCD147 were evaluated after one cycle, two cycles, and four cycles of chemotherapy. (b) The sensitivity and specificity of scMMP-9 were evaluated after one cycle, two cycles, and four cycles of chemotherapy. (c) The specificity of combined scCD147 and scMMP-9 was evaluated after one cycle, two cycles, and four cycles of chemotherapy.

Based on the changes in scCD147 and scMMP-9 serum levels, upregulation and downregulation were considered positive and negative, respectively. The changes were compared with the clinical outcomes as shown in Table 2. Then, the predictive power of scCD147 and scMMP-9 was evaluated. The sensitivity of scCD147 and scMMP-9 alone was markedly higher than the specificity after one cycle, two cycles, and four cycles of chemotherapy (Figures 3(a) and 3(b)), indicating that the upregulation of scCD147 and scMMP-9 alone was beneficial for predicting PD but not CR/PR/SD. Furthermore, when scCD147 and MMP-9 were combined into a new risk model, it had a markedly better prediction of efficacy than each protein alone, as shown in Figure 3(c). The specificity was significantly higher after one cycle, two cycles, and four cycles of chemotherapy, indicating that the combination of scCD147 and scMMP-9 could be beneficial for predicting CR/PR/SD.

### 3.5. Univariable and Multivariable Analyses for Tumor Response in NSCLC

Univariable analysis showed that variations in scMMP-9 (*P* = 0.021) after the first cycle, ECOG PS score (*P* = 0.021), regional lymph node metastasis (*P* = 0.035), variations in scCD147 (*P* = 0.002), variations in scMMP-9 (*P* = 0.001) after the second cycle, variations in scCD147 (*P* = 0.045), and variations in scMMP-9 (*P* = 0.011) after the fourth cycle were significant factors for tumor response (Table S1). In addition, multivariable analysis demonstrated that variations in scCD147 (HR: 34.29, 95% CI: 3.04-386.73; *P* = 0.004), variations in scMMP-9 (HR: 32.83, 95% CI: 3.23-333.90; *P* = 0.003) after the second cycle, and variations in scMMP-9 (HR: 12.00, 95% CI: 1.98-72.89; *P* = 0.007) after the fourth cycle may be independent factors to monitor chemotherapy outcome for patients with NSCLC (Table S2).

## 4. Discussion

Despite improvements in diagnostic and treatment methods, the prognosis of people with LC is still poor. The five-year survival of individuals with advanced LC is ≤15% [4]. Invasion and migration of tumor cells always result in tumor cells breaking through immunologic barriers such as blood and lymph [13, 14]. Studies have indicated [15–18] that several factors are involved in the invasion/metastases of tumor cells. However, the key events involved in metastases are the degradation of the basement membrane of cells and ECM [19], and MMPs play crucial roles in these cellular mechanisms [20]. Recent studies have shown that CD147 promotes MMP formation by degrading the ECM of tumor cells to promote the invasion/metastases of tumor cells [21, 22], indicating that CD147 and MMPs form an axis that might be beneficial in the prognosis of chemotherapy. Studies have already shown several promising biomarkers for the prediction of chemotherapy for patients with NSCLC, such as excision repair cross-complementing group (ERCC), thymidylate synthase (TYMS), tubulin, and ribonucleotide reductase subunit M1 (RRM1) [23]. Previously in our study, we demonstrated that changes in serum concentrations of MMP-9 in patients with NSCLC during chemotherapy were intimately associated with chemotherapy outcome [12], which was in accordance with a previous study in colorectal cancer [24]. The sensitivity of scMMP-9 was markedly higher than the specificity, indicating that the upregulation of scMMP-9 was beneficial for predicting PD. However, the specificity of scMMP-9 was insufficient in monitoring CR/PR/SD. The combination of two markers was superior to either of them alone. Here, we studied the correlation between the changes in serum concentrations of CD147/MMP-9 and chemotherapy outcome in NSCLC cases.

In recent years, several studies have shown high concentrations of CD147 expressed in laryngeal cancer [25], oral cancer [26], lung cancer [27], breast cancer [28], myeloma [29], and colon cancer [30], which indicates that CD147 serum levels may be correlated with the development, invasion, and metastasis of tumor cells [31–33]. Ding and colleagues [34] conducted a meta-analysis to assess the association of CD147 expression in patients with hepatocellular carcinoma (HCC). They found CD147 expression to be potentially closely related to survival from HCC and associated clinicopathologic parameters.

Taken together, the results of this study showed that CD147 levels were significantly higher in patients with LC than those in HVs. Serum concentrations of CD147 in patients with LC were related to distant metastases and TNM stage. We demonstrated that the serum concentrations of CD147 have promising diagnostic and predictive efficacy prospects, an observation that is in accordance with previous studies.

We carried out sequential measurements of serum concentrations of CD147 in NSCLC cases in parallel with the evaluation of chemotherapy response, similar to our previous study that focused on MMP-9 [12]. In patients with NSCLC, serum concentrations of CD147 increased markedly in those who developed PD but increased slightly or decreased in those who achieved CR/PR/SD. The difference in the alterations in serum concentrations of CD147 in patients with different chemotherapy outcomes was significantly different between the first and second chemotherapy treatments. These findings suggested that sequential variations in the serum concentrations of CD147 could be beneficial for assessing chemotherapy outcome (i.e., CR/PR/SD or PD) in people with NSCLC. Serum concentrations of CD147 that decline or stabilize suggest CR/PR/SD, but those that increase denote PD.

Several studies have shown that CD147 and MMP-9 are related to the development, invasion, and metastasis of tumor cells. Infiltration of malignant tumor cells is aided by high expression of CD147, which can increase MMP-9 activity to cause degradation of the ECM and the basement membrane of cells [35, 36]. Therefore, CD147 expression on tumor cell surfaces is, in general, considered to be increased, which leads to the stimulation of MMP-9 activity.

Similar to scMMP-9, the sensitivity of scCD147 alone was markedly higher than the specificity, and the upregulation of scCD147 alone was beneficial for predicting PD. When scCD147 and MMP-9 were combined into a new risk model, the specificity was significantly higher, indicating that the combination of scCD147 and scMMP-9 could be beneficial for predicting CR/PR/SD. These results suggested that sequential changes in the serum concentrations of CD147 and MMP-9 could be beneficial for assessing chemotherapy outcome (i.e., CR/PR/SD or PD) in patients with NSCLC.

Multivariable analysis demonstrated that variations in scCD147 and scMMP-9 after the second cycle and variations in scMMP-9 after the fourth cycle could be independent factors to monitor chemotherapy outcome for patients with NSCLC. scCD147 and scMMP-9 as a combination provide a new risk model that significantly improves the predictive power for chemotherapy response in patients with NSCLC. MMP-9 plus another protein as a combination is not a special case in our study; likewise, in colorectal cancer, scMMP-9 plus scRab1B also showed powerful predictive importance for chemotherapy outcomes [24].

Taken together, the combination of two biomarkers, CD147/MMP-9, seems to have promising benefits for the outcome of chemotherapy in patients with NSCLC. However, since the results of the aforementioned study were obtained in a retrospective single-institute study, they need to be validated in a future study involving a large number of patients in conjunction with determination of the optimal cut-off levels of biomarkers.

Serum concentrations of CD147 were (i) higher in LC cases than those in HVs, (ii) were associated with distant metastases and TNM stage in patients with LC, and (iii) may have useful diagnostic and predictive value for efficacy. Changes in serum concentrations of CD147 in patients with NSCLC during chemotherapy were intimately related to outcome. Serum concentrations of CD147 that fell or stabilized suggested CR/PR/SD, but increasing concentrations denoted PD. Variations in scCD147 and scMMP-9 may be independent factors for monitoring chemotherapy outcomes in patients with NSCLC. Furthermore, scCD147 and scMMP-9 are potential predictive biomarkers for efficacy, and their combination significantly improves the predictive power for chemotherapy response in patients with NSCLC.

## Figures and Tables

**Table 1:** The correlation between serum CD147 and MMP-9 levels before chemotherapy and clinical characteristics in patients with NSCLC.

Characteristics	CD147 (pg/mL)	*P*	MMP-9^[12]^ (*μ*g/mL)	*P*
Gender		0.959		0.121
Male	566.30 (55.88-643.03)		2.80 (1.08-6.96)	
Female	568.33 (461.50-643.03)		2.29 (0.80-3.59)	
Age		0.676		0.536
≤60	572.62 (55.88-910.80)		2.61 (0.80-5.49)	
>60	551.34 (281.00-822.25)		2.79 (1.08-6.96)	
Smoking status		0.637		0.965
Never	568.34 (176.00-643.03)		2.87 (1.34-4.84)	
Smoker	565.03 (55.88-910.80)		2.74 (0.80-6.96)	
Baseline ECOG PS		0.543		0.301
0-1	568.58 (55.88-910.80)		2.74 (0.80-6.96)	
2	554.35 (510.80-781.11)		3.66 (1.49-6.00)	
Histological subtype		0.481		0.499
Adenocarcinomas	561.25 (55.88-910.80)		2.81 (1.08-6.00)	
Squamous cell carcinomas	571.36 (281.00-822.25)		2.63 (0.80-6.96)	
T status		0.217		0.928
T1	607.60 (196.05-613.79)		2.17 (1.82-4.02)	
T2	554.35 (434.00-692.02)		2.72 (0.80-3.79)	
T3	498.33 (55.88-650.73)		2.51 (1.49-6.96)	
T4	597.16 (176.00-910.80)		2.82 (1.08-6.34)	
N status		0.423		0.800
N0	591.47 (340.00-805.80)		2.55 (1.82-4.20)	
N1	314.89 (55.88-573.90)		2.82 (2.49-3.16)	
N2	545.35 (176.00-678.60)		2.83 (1.49-4.84)	
N3	562.82 (281.00-910.80)		2.71 (0.80-6.96)	
M status		0.287		0.497
M0	551.34 (55.88-650.73)		2.72 (0.80-6.96)	
M1	569.84 (176.00-910.80)		2.78 (1.08-6.34)	
TNM stage		0.287		0.497
III	551.34 (55.88-650.73)		2.72 (0.80-6.96)	
IV	569.84 (176.00-910.80)		2.78 (1.08-6.34)	

NCSCL: non-small-cell lung cancer; ECOG: Eastern Cooperative Oncology Group; PS: performance status.

**Table 2:** The comparison of serum CD147 and MMP-9 levels alone and combined with response to chemotherapy in patients with advanced NSCLC.

Parameter	The first cycle post-chemo		Total	The second cycle post-chemo		Total	The fourth cycle post-chemo		Total
	PD	Non-PD		PD	Non-PD		PD	Non-PD	
CD147									
Positive	4	24	28	9	14	23	14	8	22
Negative	2	23	25	4	23	27	11	6	17
Total	6	47	53	13	37	50	25	14	39
MMP-9									
Positive	6	15	21	10	27	37	21	9	30
Negative	0	32	32	3	10	13	4	5	9
Total	6	47	53	13	37	50	25	14	39
CD147+MMP-9									
Positive	4	10	14	6	4	10	10	5	15
Negative	2	37	39	7	33	40	15	9	24
Total	6	47	53	13	37	50	25	14	39

## Data Availability

Answer: No. Comment: The datasets used and/or analyzed during the current study are available from the corresponding author on reasonable request.

## Abbreviations

CD147: Cluster of differentiation-147
MMP-9: Matrix metalloproteinase 9
LC: Lung cancer
ELISA: Enzyme-linked immunosorbent assay
ROC: Receiver operating characteristic
HVs: Healthy volunteers
NSCLC: Non-small-cell lung cancer
CR: Complete response
PR: Partial response
SD: Stable disease
PD: Progressive disease
SCLC: Small cell lung cancer
MMPs: Matrix metalloproteinases
ECM: Extracellular matrix
ECOG: Eastern Cooperative Oncology Group
PS: Performance status
SCC: Squamous cell carcinoma
TNM: Tumor, node, and metastases.

## Additional Points

*Key Points*. Changes in the serum concentrations of CD147 in patients with NSCLC during chemotherapy were intimately related to outcome. Serum concentrations of CD147 that fell or stabilized suggested CR/PR/SD, but increasing concentrations denoted PD. scCD147 and scMMP-9 are potential predictive biomarkers for efficacy, and their combination significantly improves the predictive power for chemotherapy response in patients with NSCLC.

## Supplementary Materials

Supplementary Materials Table S1: univariable analyses for tumor response in NSCLC cases after the various cycles of chemotherapy. Table S2: multivariable analyses for tumor response in NSCLC cases after the various cycles of chemotherapy.
