# Supplementary material for: The Combination of CD147 and MMP-9 Serum Levels Is Identified as Novel Chemotherapy Response Markers of Advanced Non-Small-Cell Lung Cancer
Source: Dis Markers. 2020 Apr 24;2020:8085053. doi: 10.1155/2020/8085053 (PMC7196144; doi:10.1155/2020/8085053)
Supplement: Supplementary Materials — Table S1: univariable analyses for tumor response in NSCLC cases after the various cycles of chemotherapy. Table S2: multivariable analyses for tumor response in NSCLC cases after the various cycles of chemotherapy. [file 8085053.f1.zip › 8085053-Supplementary material -1.docx]

**Supplementary material**

**Table S1 Univariable analyses for tumor response in NSCLC cases after the various cycles of chemotherapy**

| Variable | After the first cycle | |  | After the second cycle | |  | After the fourth cycle | |
| --- | --- | --- | --- | --- | --- | --- | --- | --- |
|  | HR (95%CI) | *P* |  | HR (95%CI) | *P* |  | HR (95%CI) | *P* |
| Gender | 0.58(0.06-5.51) | 0.638 |  | 2.04(0.39–10.85) | 0.404 |  | 2.22(0.51-9.65) | 0.286 |
| Male |  |  |  |  |  |  |  |  |
| Female |  |  |  |  |  |  |  |  |
| Age(years) | 1.61(0.29-8.86) | 0.584 |  | 0.63(0.18-2.28) | 0.483 |  | 0.43(0.11-1.76) | 0.242 |
| ≤60 |  |  |  |  |  |  |  |  |
| ＞60 |  |  |  |  |  |  |  |  |
| Smoking status | 0.74(0.08-7.08) | 0.794 |  | 1.77(0.33-9.52) | 0.507 |  | 3.94(0.88-17.73) | 0.074 |
| Never |  |  |  |  |  |  |  |  |
| Smoker |  |  |  |  |  |  |  |  |
| Baseline ECOG PS | 2.15(0.20-23.21) | 0.528 |  | 0.59(0.03-1.30) | 0.021 |  | 1.13(0.31-3.45) | 0.783 |
| 0-1 |  |  |  |  |  |  |  |  |
| ≥2 |  |  |  |  |  |  |  |  |
| T status | 0.47(0.08-2.60) | 0.386 |  | 1.30(0.35-4.84) | 0.693 |  | 0.63(0.16-2.42) | 0.499 |
| T1+ T2 |  |  |  |  |  |  |  |  |
| T3+ T4 |  |  |  |  |  |  |  |  |
| N status | 2.48(0.91-14.31) | 0.690 |  | 0.76(0.04-3.22) | 0.035 |  | 0.70(0.13-3.70) | 0.673 |
| N0 |  |  |  |  |  |  |  |  |
| N1+ N2+ N3 |  |  |  |  |  |  |  |  |
| M status | 1.35(0.14-12.92) | 0.794 |  | 0.66(0.12-3.60) | 0.630 |  | 2.48(0.25-24.65) | 0.439 |
| M0 |  |  |  |  |  |  |  |  |
| M1 |  |  |  |  |  |  |  |  |
| TNM stage | 1.35(0.14-12.92) | 0.794 |  | 0.66(0.12-3.60) | 0.630 |  | 2.48(0.25-24.65) | 0.439 |
| Ⅲ |  |  |  |  |  |  |  |  |
| Ⅳ |  |  |  |  |  |  |  |  |
| variations of CD147 | 0.23(0.04-1.42) | 0.115 |  | 14.85(2.79-70.06) | 0.002 |  | 3.20(0.82-12.53) | 0.045 |
| increased |  |  |  |  |  |  |  |  |
| decreased |  |  |  |  |  |  |  |  |
| variations of MMP-9 | 6.51(0.98-20.18) | 0.021 |  | 14.29(3.09-65.99) | 0.001 |  | 7.00(1.56-31.52) | 0.011 |
| increased |  |  |  |  |  |  |  |  |
| decreased |  |  |  |  |  |  |  |  |
