# Supplementary material for: The Combination of CD147 and MMP-9 Serum Levels Is Identified as Novel Chemotherapy Response Markers of Advanced Non-Small-Cell Lung Cancer
Source: Dis Markers. 2020 Apr 24;2020:8085053. doi: 10.1155/2020/8085053 (PMC7196144; doi:10.1155/2020/8085053)
Supplement: Supplementary Materials — Table S1: univariable analyses for tumor response in NSCLC cases after the various cycles of chemotherapy. Table S2: multivariable analyses for tumor response in NSCLC cases after the various cycles of chemotherapy. [file 8085053.f1.zip › 8085053-Supplementary material-2.docx]

**Supplementary material**

**Table S2 Multivariable analyses for tumor response in NSCLC cases after the various cycles of chemotherapy**

| Variable | After the first cycle | |  | After the second cycle | |  | After the fourth cycle | |
| --- | --- | --- | --- | --- | --- | --- | --- | --- |
|  | HR (95%CI) | *P* |  | HR (95%CI) | *P* |  | HR (95%CI) | *P* |
| Baseline ECOG PS |  |  |  | 14.29(3.09-35.98) | 0.285 |  |  |  |
| 0-1 |  |  |  |  |  |  |  |  |
| ≥2 |  |  |  |  |  |  |  |  |
| N status |  |  |  | 0.32(0.00-1.51) | 0.138 |  |  |  |
| N0 |  |  |  |  |  |  |  |  |
| N1+ N2+ N3 |  |  |  |  |  |  |  |  |
| variations of CD147 |  |  |  | 34.29(3.04-386.73) | 0.004 |  | 5.51(0.98-30.88) | 0.052 |
| increased |  |  |  |  |  |  |  |  |
| decreased |  |  |  |  |  |  |  |  |
| variations of MMP-9 | 0.000 | 0.997 |  | 32.83(3.23-333.90) | 0.003 |  | 12.00(1.98-72.89) | 0.007 |
| increased |  |  |  |  |  |  |  |  |
| decreased |  |  |  |  |  |  |  |  |
