# Supplementary material for: The Combination of CD147 and MMP-9 Serum Levels Is Identified as Novel Chemotherapy Response Markers of Advanced Non-Small-Cell Lung Cancer
Source: Dis Markers. 2020 Apr 24;2020:8085053. doi: 10.1155/2020/8085053 (PMC7196144; doi:10.1155/2020/8085053)
Supplement: Supplementary Materials — Table S1: univariable analyses for tumor response in NSCLC cases after the various cycles of chemotherapy. Table S2: multivariable analyses for tumor response in NSCLC cases after the various cycles of chemotherapy. [file 8085053.f1.zip › mat.8085053.v2.pdf]

Supplementary material

Table S1 Univariable analyses for tumor response in NSCLC cases after the various cycles of chemotherapy

Variable	After the first cycle		After the second cycle		After the fourth cycle	
	HR (95%CI)	*P*	HR (95%CI)	*P*	HR (95%CI)	*P*
Gender	0.58(0.06-5.51)	0.638	2.04(0.39–10.85)	0.404	2.22(0.51-9.65)	0.286
Male						
Female						
Age(years)	1.61(0.29-8.86)	0.584	0.63(0.18-2.28)	0.483	0.43(0.11-1.76)	0.242
≤60						
＞60						
Smoking status	0.74(0.08-7.08)	0.794	1.77(0.33-9.52)	0.507	3.94(0.88-17.73)	0.074
Never						
Smoker						
Baseline ECOG PS	2.15(0.20-23.21)	0.528	0.59(0.03-1.30)	0.021	1.13(0.31-3.45)	0.783
0-1						
≥2						
T status	0.47(0.08-2.60)	0.386	1.30(0.35-4.84)	0.693	0.63(0.16-2.42)	0.499
T1+ T2						
T3+ T4						
N status	2.48(0.91-14.31)	0.690	0.76(0.04-3.22)	0.035	0.70(0.13-3.70)	0.673
N0						
N1+ N2+ N3						
M status	1.35(0.14-12.92)	0.794	0.66(0.12-3.60)	0.630	2.48(0.25-24.65)	0.439
M0						
M1						
TNM stage	1.35(0.14-12.92)	0.794	0.66(0.12-3.60)	0.630	2.48(0.25-24.65)	0.439
III						
IV						
variations of CD147	0.23(0.04-1.42)	0.115	14.85(2.79-70.06)	0.002	3.20(0.82-12.53)	0.045
increased						
decreased						
variations of MMP-9	6.51(0.98-20.18)	0.021	14.29(3.09-65.99)	0.001	7.00(1.56-31.52)	0.011
increased						
decreased						

Table S2 Multivariable analyses for tumor response in NSCLC cases after the various cycles of chemotherapy

Variable	After the first cycle		After the second cycle		After the fourth cycle	
	HR (95%CI)	*P*	HR (95%CI)	*P*	HR (95%CI)	*P*
Baseline ECOG PS			14.29(3.09-35.98)	0.285		
0-1						
≥2						
N status			0.32(0.00-1.51)	0.138		
N0						
N1+ N2+ N3						
variations of CD147			34.29(3.04-386.73)	0.004	5.51(0.98-30.88)	0.052
increased						
decreased						
variations of MMP-9	0.000	0.997	32.83(3.23-333.90)	0.003	12.00(1.98-72.89)	0.007
increased						
decreased						
